# Roles and Mechanisms of Deubiquitinases (DUBs) in Breast Cancer Progression and Targeted Drug Discovery

**DOI:** 10.3390/life11090965

**Published:** 2021-09-14

**Authors:** Sixuan Li, Hongquan Zhang, Xiaofan Wei

**Affiliations:** Department of Human Anatomy, Histology and Embryology, School of Basic Medical Sciences, Peking Unversity Health Science Center, Beijing 100191, China; sicons@pku.edu.cn (S.L.); hongquan.zhang@bjmu.edu.cn (H.Z.)

**Keywords:** deubiquitinase, breast cancer, tumor growth and metastasis, cancer therapy

## Abstract

Deubiquitinase (DUB) is an essential component in the ubiquitin—proteasome system (UPS) by removing ubiquitin chains from substrates, thus modulating the expression, activity, and localization of many proteins that contribute to tumor development and progression. DUBs have emerged as promising prognostic indicators and drug targets. DUBs have shown significant roles in regulating breast cancer growth, metastasis, resistance to current therapies, and several canonical oncogenic signaling pathways. In addition, specific DUB inhibitors have been identified and are expected to benefit breast cancer patients in the future. Here, we review current knowledge about the effects and molecular mechanisms of DUBs in breast cancer, providing novel insight into treatments of breast cancer-targeting DUBs.

## 1. Introduction

**Figure 1:**
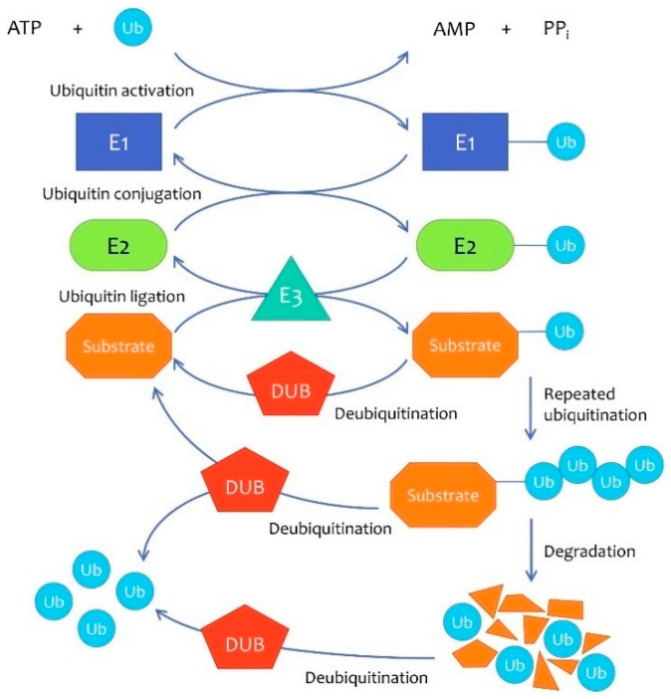
The ubiquitin–proteasome degradation pathway. The ubiquitin–proteasome system (UPS) is one of the protein degradation pathways in eukaryotic cells. Ubiquitin (Ub) is activated by E1 in an ATP-dependent manner and then transferred to E2. E3 recognizes the specific substrate protein and catalyzes the activated Ub transferring from E2 to substrate. In addition, proteins with Lys48-linked polyubiquitin chains are usually degraded by the 26s proteasome. At the same time, the deubiquitinases (DUBs) counteract the E3s and reverse the process by cleaving the isopeptide bonds, thus protecting these proteins from degradation and improving Ub recycling.

The ubiquitin–proteasome system (UPS) is one of the protein degradation pathways in eukaryotic cells. Ubiquitin (Ub), a highly conserved regulatory protein, is conjugated to protein substrates by ubiquitin-activating enzymes (E1s), ubiquitin-conjugating enzymes (E2s), and ubiquitin-ligating enzymes (E3s), successively [1]. Conversely, deubiquitinases (DUBs) remove ubiquitin chains from substrates by specifically cleaving the isopeptide bond or the peptidic bond [2] (Figure 1).

**Figure 2:**
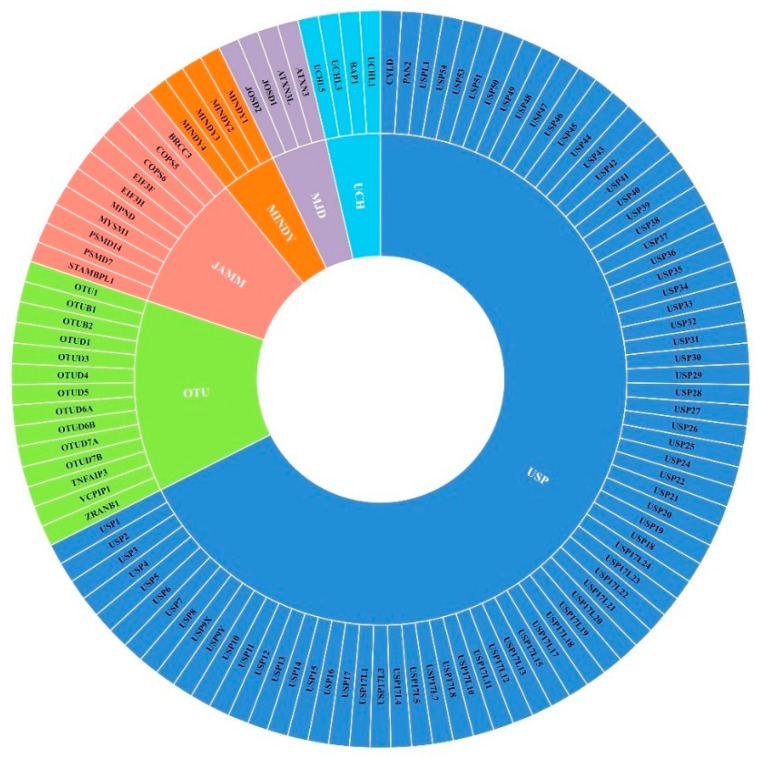
Classification of DUBs. DUBs are structurally classified into six families: USPs (ubiquitin-specific proteases), UCHs (ubiquitin carboxy-terminal hydrolases), MJDs (Machado–Josephin domain-containing proteases), OTUs (ovarian tumour proteases), MINDYs (motif-interacting with ubiquitin-containing novel DUB family), and JAMMs (JAB1/MPN/MOV34 family).

The human genome encodes approximately 100 DUBs that can be classified into six families structurally: USPs (ubiquitin-specific proteases), UCHs (ubiquitin carboxy-terminal hydrolases), MJDs (Machado–Josephin domain-containing proteases), OTUs (ovarian tumor proteases), MINDYs (motif-interacting with ubiquitin-containing novel DUB family), and JAMMs (JAB1/MPN/MOV34 family) [3] (Figure 2). DUBs play important roles in protein homeostasis, and regulate multiple biological processes and signaling pathways involved in tumor initiation and progression, such as cell-cycle regulation, DNA damage repair, chromatin remodeling, and the transforming growth factor-β (TGF-β) pathway [2].

As the most common malignancy in women, breast cancer is in urgent need of novel drugs and strategies to improve curative efficiencies and reduce toxicities [4]. As DUBs have been demonstrated to exert profound effects in tumors and could be used as drug targets in cancer therapeutics, more and more studies focus on how DUBs function in breast cancer progression and treatment. Previously, a review published in 2016 by Xiao and colleagues suggested breast cancer-promoting DUBs, breast cancer-suppressing DUBs, and strategies to develop DUB inhibitors. The authors mainly focused on the roles of several important DUBs in breast cancer, such as USP9X, USP15, and CYLD(Cylindromatosis tumor suppressor protein). In this review, we summarize the past five years of results, and go a step further by discussing how DUBs function in every stage of breast cancer progression, including tumor growth, tumor metastasis, immunosuppression, chemoresistance, and radioresistance in breast cancer. The therapeutic potential for the pharmacological modulation of DUB activities is also discussed.

## 2. DUBs in Breast Cancer Growth

### 2.1. DUBs of c-Myc

Myc-family proteins act as central factors in cell proliferation and tumor initiation pathways [5], and only a few DUBs control the activity and stability of c-myc. For example, a recent study showed that USP5 upregulates β-catenin and its downstream signals including c-Myc in trophoblast cells [6]. It was reported that USP22 and USP36 promote breast cancer growth targeting the oncogenic protein, c-Myc.

USP22, a member of the cancer stem cell (CSC) signature, is required for Myc-driven transcription [7]. USP22 stabilizes c-Myc by removing the poly-ubiquitin chains and antagonizing the ubiquitination activity of its E3 ligase, FBW7, and consequently promoting breast cancer cell growth [8].

USP36 contributes to breast tumorigenesis by forming a positive feedback loop with c-Myc. USP36 increases breast cancer cell proliferation by deubiquitinating c-Myc in the nucleolus and inhibiting c-Myc proteasomal degradation mediated by SCF^Fbw7^, reciprocally, Myc targets USP36 for promoting its transcription [9].

### 2.2. DUBs of KLF5

The transcriptional regulator, Krüppel-like factor 5 (KLF5), performs protumorigenic activity in breast cancer via interacting with critical survival pathways [10]. High expression of KLF5 is observed in ER^-^/PR^-^/CK5^+^ breast cancer cell lines [11], and is regarded as a poor prognosis factor in breast cancer [12]. Importantly, KLF5 represents a novel target for triple-negative breast cancer (TNBC) treatment [13]. Scientists have identified several DUBs which deubiquitinate and stabilize KLF5, including ATXN3L, BAP1, and USP3. As expected, these DUBs promote breast cancer development partly via KLF5 [13,14,15]. At the same time, BAP1 is downregulated by miR-125a-5p, which functions as a tumor suppresser and is abnormally expressed in breast cancer [16].

### 2.3. DUBs That Regulate H2B Monoubiquitination Levels

H2B monoubiquitination (H2Bub1) plays a tumor-suppressing role in breast cancer, and its level becomes absent during tumor progression [17].

The human SAGA complex (hSAGA) is an essential regulator of H2Bub1 levels [7]. It is believed that imbalances of USP22, USP27X, and USP51 lead to SAGA-related breast cancer development [18]. USP22 has been reported as a catalytic subunit of hSAGA that deubiquitinates histone H2b with the help of the regulatory components, ATXN7L3 and ENY2 [7,18]. USP27X and USP51, independent of hSAGA structurally, deubiquitinate histone H2B by competing with USP22 to interact with the regulatory components. Depletion of USP22, USP51, or USP27X inhibits breast cancer growth partly through downregulation of H2Bub1 [18].

USP44 is another deubiquitinase modulating H2Bub1, which suppresses or promotes breast cancer growth determined by the particular subtypes of breast cancer. H2Bub1 restricts tumor development in basal-like cancers and its level tends to be low in such tissues, while H2Bub1 shows promotive effects in luminal subtypes. Thus, USP44 exerts opposing effects by removing ubiquitin from H2Bub1 [19].

### 2.4. DUBs of Cell Cycle Regulatory Components

The disorder of the cell cycle machinery leads to abnormal cell proliferation, which is regarded as the hallmark of tumor initiation [20]. During the cell cycle, several regulatory components promote or impede transitions between different phases.

Apart from facilitating c-Myc-driven transcription, USP22 also increases breast cancer cell proliferation partly through its nontranscriptional activity on regulating the cell cycle machinery and promoting the G1-S transition. USP22 deubiquitinates and protects G1 cyclin D1 (CCND1) from proteasome-mediated degradation, which regulates G1-S progression through activating CDK4 and CDK6, and thus functions as a cellular proto-oncogene primarily [21]. In addition, USP22 is identified as a biomarker in ER^+^ breast cancer by enhancing tumor growth via stabilization of ERα [22].

USP14 plays a critical role in multiple kinds of tumors through modulating cell proliferation, migration, and autophagy [23]. Elevated expression of USP14 has been observed in breast cancer tissues and the level of USP14 is inversely relevant to long-term survival [24]. In vitro, knockdown of USP14 restrains cancer cell proliferation via apoptosis induction and cell cycle arrest in G_2_/M phase. Mechanistically, USP14 controls cell cycle progression through deubiquitination of CyclinB1, which functions as a crucial indicator predictive of the survival in ER^+^ breast cancer [25,26].

### 2.5. Other DUBs in Breast Cancer Growth

USP7 has been demonstrated as an oncoprotein in many tumors by deubiquitinating numerous substrates involved in multiple typical pathways [27]. Reports have shown that USP7 overexpression is an indicator of poor prognosis for breast cancer patients. ERα is deubiquitinated and stabilized by USP7, which in turn promotes cell proliferation and tumor growth in ERα^+^ breast cancer through inhibiting cell cycle arrest and apoptosis [28]. Another novel substrate of USP7 is a histone demethylase PHF8, whose upregulation was shown to be involved in the development and progression of gastric cancer and other malignant tumors [29,30]. Researchers proposed that USP7 forms a positive oncogenic feedback loop with PHF8. PHF8 is stabilized by USP7 through deubiquitination, resulting in increased expression of cyclin A2, which promotes the proliferation of breast cancer cells and accelerates tumor growth. At the same time, PHF8 contributes to the abundance of USP7 in breast cancer by transcriptionally facilitating its encoding genes [31]. ECT2, which is deubiquitinated and stabilized by USP7, was found to enhance breast cancer cell proliferation in vitro and cancer growth in vivo by positively regulating the classical oncogenic signaling axis mediated by MDM2. Importantly, USP7 is able to interact with itself and remove its own polyubiquitin chains to sustain self-stability. In addition, this process is observed to be promoted by ECT2, indicating USP7 and ECT2 also construct a positive feedback loop similar to the USP7–PHF8 loop [32].

Breast tumor initiating cells (BTICs) exert important effects on tumor initiation [33]. Elevated expression of Interleukin-1 receptor type 2 (IL1R2) is demonstrated in the BTIC population, and is relevant to poor prognosis in breast cancers. Consistently, IL1R2 enhances breast cancer cell proliferation in vitro, and facilitates xenograft tumor growth in vivo. The deubiquitinase USP15 is a downstream target of IL1R2 [34]. IL1R2 intracellular domain (icd-IL1R2) interacts with USP15, enhancing its deubiquitination and stabilization of a cell cycle regulator and tumor growth promoter, BMI1 [34,35].

OTUD3 exhibits tumor-promoting activity in lung cancer [36], and conversely shows tumor-suppressing activity in breast cancer. OTUD3 rescues p53 from proteasomal degradation regulated by MDM2, thus activating cancer cell apoptosis in vitro [37]. OTUD3 also stabilize PTEN by removing ubiquitin linkages from PTEN and binding to its C2 domain. Additionally, various loss-of-function mutations and decreased expression of OTUD3 have been reported in malignancies, indicating its clinical significance in breast cancer [38].

USP4 contextually shows paradoxical tumor-promoting and -suppressing effects in breast cancer growth. A decrease in USP4 expression is found in breast cancer tissues, indicating a tumor-suppressive role for USP4. Correspondingly, USP4 inhibits ubiquitin–proteasome-dependent degradation of PDCD4, hindering cancer cell proliferation in vitro [39]. In contrast, the TRPS1–USP4–HDAC2 regulatory axis performs an oncogenic function in breast cancer growth. USP4 is recruited by TRPS1 scaffolding to deubiquitinate HDAC2, which represses activity of antigrowth genes on the transcriptional level, thus leading to an acceleration of cell proliferation [40].

USP9X is an important regulator in tumorigenesis targeting multiple signaling pathways. USP9X is overexpressed in breast cancer tissues, promoting tumor growth through the regulation of the cell cycle [41]. Interestingly, USP9X enhances the stability of a E3 ubiquitin ligase, RNF115, which has also been shown to promote cell proliferation in ERα^+^ breast cancers via downregulation of p21 [42,43]. Moreover, the canonical Wnt signaling pathway dysregulation is frequently observed in cancer development [44]. BCL9, a coactivator for Wnt/β-catenin transcription, is shown to be a substrate for USP9X. Deubiquitination of BCL9 reinforces the construction of β-catenin/BCL9/PYGO complex, which in turn activates promoters of Wnt target genes and facilitates breast cancer carcinogenesis [45]. In addition, USP9X regulates centrosome duplicating via deubiquitination and stabilization of CEP131 in centrosome. The USP9X–CEP131 axis is involved in centrosome amplification and mitotic defects, and consequently promotes breast carcinogenesis [46]. Investigators also revealed the role of USP9X in regulating the Hippo–YAP pathway. YAP1 is deubiquitinated and stabilized by USP9X, accelerating cell proliferation in breast cancers [47]. Intriguingly, USP9X suppresses tumor growth by deubiquitinating and potentiating LATS kinase, a component in the Hippo pathway. LATS kinase phosphorylates and inactivates YAP/TAZ, whose activation form is supposed to interact with TEAD and prompt transcription of downstream pro-proliferative and antiapoptotic genes, respectively [48].

The overexpression of EZH2 promotes tumorigenesis in many kinds of tumors. ZRANB1, a seldom-studied DUB, was identified as a novel EZH2 deubiquitinase, which stabilizes EZH2 through its OTU domain, thus enhancing breast cancer development [49].

CYLD, which was originally identified in familial cylindromatosis, contains a catalytic domain of DUB [50]. CYLD expression is repressed in breast cancer tissues and functions as an independent prognostic index [51]. CYLD inhibits breast cancer growth by negatively modulating the NF-*κ*B pathway and targeting several regulators of NF-*κ*B via deubiquitinase activity [52]. For example, CYLD removes ubiquitin chains from TRAF2, which is necessary for activating IKK, a central component to sustain NF-*κ*B signaling [53]. Thus, inhibition of CYLD promotes breast tumorigenesis via activation of NF-κB signaling. MicroRNAs including miR370-3p and miR-301b are identified in vivo to strengthen breast cancer growth through downregulation of CYLD [54,55]. Specifically, miR-301b plays an oncogenic role in TNBC partly via reduction in CYLD [55].

The deubiquitinase PSMD2 is a noncatalytic subunit of the 19S regulatory complex of the 26s proteasome [56]. Researchers have found that PSMD2 is significantly overexpressed in breast cancer and is related to poor clinical outcomes. PSMD2 increases cell proliferation and inhibits cell cycle arrest in vitro and promotes tumor growth in vivo. Mechanistically, PSMD2, with the assistance of another deubiquitinase USP14, interacts with CDK inhibitors p21 and p27, constraining their ubiquitin–proteasome degradation [57].

USP21 has been demonstrated as a key DUB in sustaining cancer stem cell properties [58]. USP21 stabilizes FOXM1 via deubiquitination, which functions as a transcription factor in driving cell cycle progression, thus increasing proliferation of basal-like breast cancer (BLBC) cells. According to databases, USP21 is generally overexpressed in patients with BLBC, positively correlating with the increase in FOXM1 [59].

USP28, a therapeutic target for many tumors [60], promotes breast cancer growth in vivo through deubiquitination and stabilization of lysine-specific demethylase 1 (LSD1), which is essential in sustaining the pluripotency of embryonic stem cells [61,62]. It is found that knockdown of USP28 induces differentiation and suppresses self-renewal in breast cancer stem cells (CSCs) by elevating expression of differentiation genes and decreasing expression of pluripotent molecules [61]. At the same time, histone deacetylase 5 (HDAC5) enhances USP28 stability and facilitates breast cancer cell proliferation in a LSD1-dependent manner [63]. Thus, the HDAC5–USP28–LSD1 axis plays an important role in promoting breast tumorigenicity.

## 3. DUBs in Breast Cancer Metastasis

### 3.1. TGF-β Signaling Pathway

Dysregulation of the transforming growth factor-β (TGF-β) signaling pathway is crucial in tumor progression, therefore, TGF-β inhibition is used systematically in several kinds of tumors. TGF-β signaling promotes metastasis by enhancing cell motility, invasion, EMT, and creating a favorable microenvironment for cancer cell engraftment and growth in metastatic sites [64].

**Figure 3:**
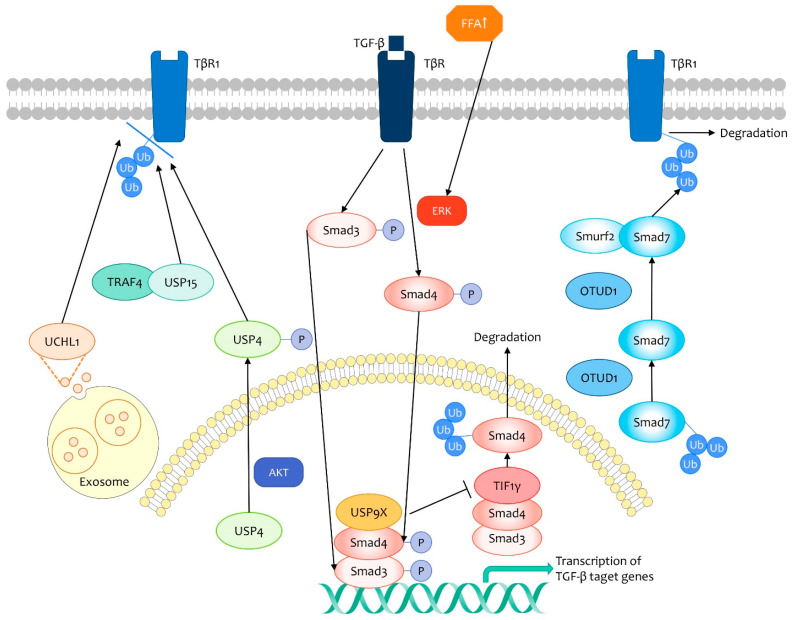
The role of DUBs in TGF-β-mediated breast cancer metastasis. DUBs regulate the TGF-β pathway to function in breast cancer metastasis. UCHL1. USP15 and USP4 stabilize membrane TGF-β type I receptor (TβRI) via deubiquitination, and thus promote TGF-β-induced metastasis. UCHL1 is released by exosomes. USP15 is recruited to TβRI with the assistance of TRAF4. USP4 is phosphorylated by AKT, which promotes its migration from nucleus to cytoplasm and membrane. In contrast, OTUD1 downregulates TβRI by stabilizing the SMAD7–SMUF2 complex, which constructs a E3 ubiquitin ligase targeting TβRI for degradation. Elevated free fatty acid (FFA) promotes TGF-β-induced ERK activation, which phosphorylates SMAD4 and promotes the formation of the USP9X–Smad4–Smad3 complex, enhancing transcription of downstream genes of the TGF-β pathway.

TGF-β type I receptor (TβRI) is a central component of the TGF-β pathway, improving activity of this signaling in multiple physiological processes through enhancement of stability and increase in cell membrane location. Importantly, USP4, USP15, and UCHL1 have been confirmed as critical regulators in the TGF-β pathway targeting membrane TβRI for deubiquitination and stabilization [65,66,67]. During this process, USP4 is phosphorylated by AKT, inducing its translocation from nucleus to cytoplasm and membrane, and preserves its deubiquitinase activity for TβRI [65]. In breast cancer cells, USP15 is recruited to TβRI with the assistance of TRAF4, a E3 ubiquitin ligase that also blocks SMURF2 inhibitory effects towards TGF-β pathway via degradation [68]. Additionally, relatively elevated UCHL1 expression have been observed in sera exosomes of TNBC patients, suggesting that UCHL1 exerts effects on metastasis through a paracrine pathway [67]. As a result, AKT–USP4, TRAF4–USP15, and UCHL1 promote breast cancer invasion and metastasis mediated by the TGF-β pathway (Figure 3).

In contrast, OTUD1 suppresses breast cancer metastasis via downregulation of membrane TβRI [69]. SMAD7 is deubiquitinated by OTUD1 at Lysine 220 with the exposure of its PY motif, which subsequently interacts with SMURF2. The SMAD7–SMUF2 complex constructs a E3 ubiquitin ligase, and is recruited to the cell surface, inducing the lysosomal and proteasomal degradation of TβRI [69,70] (Figure 3).

Obesity is an important risk factor for distant failures and poor survival rates in breast cancers. Recently, the mechanism of how obesity intensifies TGF-β-mediated invasion has been revealed. Elevated free tatty acid (FFA) promotes ERK activation induced by TGF-β and consequently phosphorylates SMAD4 at Thr277, enhancing USP9X selective interaction with SMAD4. USP9X prohibits TIF1γ, a E3 ubiquitin ligase, from interacting with SMAD4. As a result, USP9X deubiquitinates SMAD4 and promotes its reservation at the cell nucleus, where SMAD4 binds to SMAD3, and targets transcription of downstream genes in TGF-β pathway [71] (Figure 3).

### 3.2. DUBs That Target EMT Regulators

During the process of epithelial–mesenchymal transition (EMT), static epithelial cells reconstruct their cytoskeleton with the loss of cell polarity, then transdifferentiate into migratory mesenchymal cells [72]. Several studies have suggested that EMT is essential in tumor metastasis through improvement of cell mobility and resistance to apoptosis [73].

**Figure 4:**
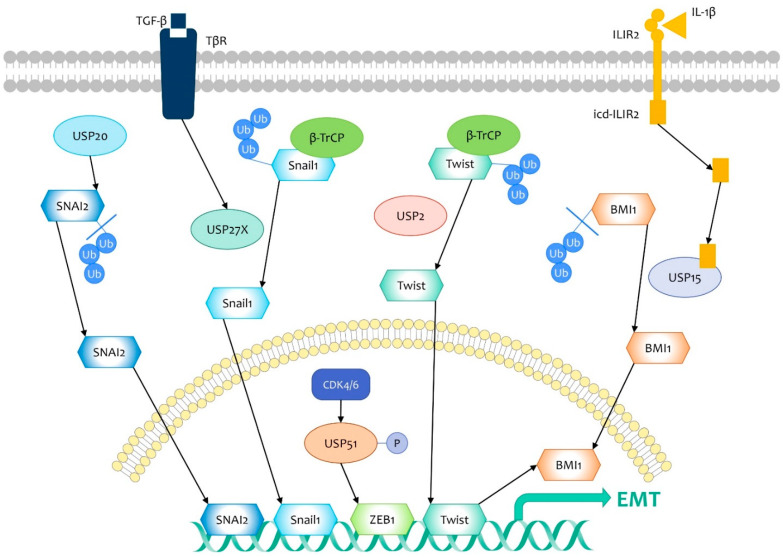
DUBs targeting EMT regulators in breast cancer metastasis. DUBs regulate epithelial–mesenchymal transition (EMT)-induced breast cancer metastasis through targeting EMT inducers, including Snail1, SNAI2, ZEB1, Twist, and BMI1. USP27 is upregulated by TGF-β, and stabilizes Snail1 against β-TrCP. USP20 stabilizes SNAI2 via deubiquitination. USP51, phosphorylated by CDK4/6, deubiquitinates ZEB1. USP2 stabilizes Twist against β-TrCP, promoting the transcription of BMI1. USP15, in cooperation with ILIR2, deubiquitinates and stabilizes BMI1. Therefore, these DUBs promote breast cancer metastasis by stabilizing EMT inducers.

Several master regulators drive EMT through repressing epithelial markers and activating genes that induce cells to acquire mesenchymal properties [72]. Among them, overexpression of the Snail/Slug family is related with poor outcomes in breast cancer patients. Multiple signaling pathways are involved in the initiation and promotion of EMT by activating Snail, including the TGF-β pathway [74]. USP27X, positively regulated by TGF-β, has been reported as a highly active deubiquitinase stabilizing Snail1 (Figure 4). Knockdown of USP27X impedes cell invasion and tumor metastasis induced by Snail1. In addition, Snail1 significantly correlates with USP27X in TNBC patients, suggesting its promotive role in an aggressive subtype [75]. Another study discovered that SNAI2 is deubiquitinated and stabilized by USP20, increasing cell migration and invasion in vitro and lung colonization in vivo (Figure 4). USP20 positively correlates with SNAI2 in breast cancer patients and a high level of USP20 is suggestive of adverse outcomes in ER^-^ breast cancers [76].

The zinc-finger E-box-binding (Zeb) family is also a master promoter in EMT. USP51 has been demonstrated as a deubiquitinase of ZEB1 in breast cancer cells, and its catalytic activity is induced by phosphorylation of CDK4/6 (Figure 4). Therefore, the CDK4/6–USP51–ZEB1 axis enhances EMT-mediated metastasis in breast cancer, providing a new target for advanced breast cancer management [77].

BMI1 is regarded as an EMT inducer [78]. As noted above, ILIR2 expression is relevant to the properties of BTICs, promoting cancer cell migration and invasion through deubiquitinating and stabilizing BMI1 in cooperation with USP15 [34]. Furthermore, USP2 stabilizes twist against β-TrCP-prompted degradation, and thus enhances transcription of BMI1. In TNBC cells, USP2 inhibition decreases Twist/BMI1-promoted cell migration (Figure 4). Based on clinical data of breast cancer patients, USP2 expression shows a positive correlation with the stage of lymph node metastasis [79].

### 3.3. Other DUBs Regulating Breast Cancer Metastasis

Overexpression of USP22 has been frequently observed in patients with invasive breast cancer, relating to fast progression and adverse outcomes in breast cancer [80]. Mechanistically, USP22 enhances cell migration and tumorigenesis in breast cancer partially through its deubiquitination and stabilization of c-Myc [8].

Apart from enhancing cell proliferation, KLF5 also promotes breast cancer metastasis [81]. However, among the three DUBs s that regulate KLF5-mediated tumor growth mentioned above, only BAP1 promotes cell migration and lung metastasis partly via upregulation of KLF5 [15]. At the same time, ATXN3 deubiquitinates and stabilizes KLF4, a closely related member of KLF5, enhancing cell migration and lung metastasis in breast cancer. Consistently, high expression of ATXN3 and KLF4 serve as indicators of an adverse prognosis in breast cancer [82].

USP9X plays an important role in promoting breast cancer metastasis. RNF115 is a novel substrate for USP9X, and the USP9X–RNF115 axis is involved in aggressive phenotypes by increasing breast cancer cell invasion and migration [42]. USP9X also facilitates cancer cell invasion induced by the Wnt/β-catenin pathway. BCL9, a component of this canonical pathway, is deubiquitinated by USP9X, and thus induces transcription of downstream genes [45].

Dysregulation of the Hippo pathway has been confirmed in multiple malignancies. EIF3H was reported to function as a novel deubiquitinase of YAP, protecting it from degradation, and the exact catalytic sites of EIF3H were identified. Therefore, EIF3H promotes breast cancer invasion and metastasis through stabilization of the Hippo/YAP signaling pathway [83].

USP1 enhances breast cancer metastasis by deubiquitinating and stabilizing KPNA2. Clinical evidence shows that USP1 expression is related to adverse outcomes in breast cancer. In addition, as expected, USP1 expression positively correlates with KPNA2 in breast cancer tissues [84].

UCHL1 enhances HIF-1 activity through deubiquitination and stabilization of its subunit HIF-1α [85]. Since HIF-1 induces vascular metastasis of breast cancer cells to the lungs, the UCHL1–HIF-1 axis promotes distant tumor metastasis, especially under hypoxic conditions [85,86]. UCHL1 expression level correlates with poor prognosis of patients with breast cancer, indicating UCHL1 as a prognostic marker and therapeutic target [85].

As mentioned above, OTUD3 is identified as a novel deubiquitinase for PTEN, a repressor in breast tumorigenesis. Intriguingly, OTUD3 increases PTEN stabilization via removing poly-ubiquitin chains, meanwhile, OTUD3 binds the PTEN C2 domain, which contributes to regulation of cell migration. As a result, the OTUD3–PTEN axis represses cell migration and tumor metastasis significantly [38].

## 4. DUBs in Immunosuppression of Breast Cancer

Although cancer cells express antigens that can be recognized by T cells and activate the immune system [87], most tumors escape from immune surveillance through various mechanisms, including selfmodification of cancer cells and alteration of tumor microenvironment. For instance, cancer cells highly express programmed death ligand 1 (PD-L1), bind with its receptor, and downregulate the activation of immune responses induced by T cells [88].

CSN5 was identified as a critical component in PD-L1-mediated immune evasion that inhibits PD-L1 poly-ubiquitination and protects it from proteasomal degradation. It is found that CSN5 is upregulated transcriptionally by NF-κB activation of p65 [89]. Additionally, lncRNA also functions as an upstream signal to regulate CSN5. LncRNA GATA3-AS1 enhances CSN5 expression via separation of miR-676-3p from CSN5, thus contributing to the immune escape of TNBC cells [90]. According to clinical evidence, the level of CSN5 is positively related with PD-L1 in breast cancer tissues, and overexpression of CSN5 indicates poor prognosis in patients with breast cancers [89].

Recently, OTUB1 has been found as a novel DUB of PD-L1 in breast cancer. OTUB1 stabilizes PD-L1 and protects it from endoplasmic reticulum-associated degradation (ERAD) by removing its K48-linked ubiquitin chains. Consistently, loss of OTUB1 leads to PD-L1 reduction in breast cancer cells, enhancing their sensitivity to the cytotoxicity of immune cells [91].

In conclusion, the NF-κB/p65/CSN5/PD-L1, GATA3-AS1/miR-676-3p/CSN5/PD-L1, and OTUB1/PD-L1 axis promotes the immunosuppression of breast cancer.

## 5. DUBs in Chemoresistance and Chemosensitivity of Breast Cancer

The high incidence of breast cancer patients relapsing after chemotherapy indicates that breast cancer cells have complex mechanisms of chemoresistance.

### 5.1. Tamoxifen (SERM)

Generally, breast cancer is an estrogen-dependent malignancy. Consequently, chemotherapy with tamoxifen, a representative drug of estrogen antagonists, possesses a good therapeutic effect on patients with breast cancer, and changes in the ERα signaling pathway intensify the tendency of endocrine resistance [92].

USP22 deubiquitinates and stabilizes ERα, enhancing ERα-induced transactivation in breast cancer cells. At the molecular level, USP22 is demonstrated as a coactivator of downstream genes, which interacts with the *cis*-acting element together with ERα. As a result, USP22 increases breast cancer resistance to ERα antagonists. In breast cancer cell lines, USP22 reduction enhances the inhibitory effects on proliferation of ERα antagonist ICI 182,780 and tamoxifen by increasing cell sensitivity to endocrine therapy [22].

USP1 is also an essential deubiquitinase in ERα signaling, which enhances ERα stability through cleaving its Lys48-linked ubiquitin chains. According to TCGA and KMPLOT databases, high expression of USP1 is relevant to poor prognosis in ERα^+^ breast cancer patients [93].

Knockdown of USP9X gives rise to tamoxifen resistance by enhancing ERα’s interaction with chromatin. Although there is a physical interaction between USP9X and ERα, ERα is not the direct substrate for USP9X, indicating USP9X may deubiquitinate ERα cofactors to regulate ERα binding with chromatin [94].

It is found that the epidermal growth factor receptor (EGFR) represses ERα transcription via hyperactivation of MAPK signaling [95]. In addition, UCHL1 downregulates ERα by deubiquitinating and stabilizing EGFR, thus increasing tamoxifen resistance in ERα^-^ breast cancer. UCHL1 inhibition offers a novel treatment for breast cancer patients with ERα shortage and decrease [96].

### 5.2. Enzalutamide (Antiandrogen)

According to the results of tissue microarrays from 3093 patients, 77% invasive breast carcinomas are androgen receptor (AR) positive, indicating AR is frequently expressed in breast tumors [97]. The AR pathway is critical in AR^+^ breast cancer, functionally interacting with multiple classic oncogenic signaling pathways. Importantly, AR-targeted therapies, including the AR antagonist, enzalutamide, have been demonstrated to be effective against breast cancer [98]. USP14 is required for enhancing AR^+^ breast cancer cell proliferation through deubiquitination and stabilization of AR [99]. Moreover, USP14 expression has a positive correlation with AR expression according to the results from the TCGA database, and is remarkably high in all subtypes of breast cancer. Thus, USP14 promotes resistance to enzalutamide in AR^+^ breast cancer [100].

### 5.3. Genotoxic Agents

Genotoxic agents such as doxorubicin (Dox) [101], irinotecan (CPT-11) [102], and cisplatin [103], are regarded as conventional treatments for breast cancer patients.

OTULIN, a member of OTU family, selectively recognizes and removes linear polyubiquitin chains from proteins [104]. OTULIN enhances TNBC resistance to Dox and CPT-11 through activation of the Wnt/β-catenin pathway, which contributes to chemoresistance by maintaining CSCs. Mechanistically, DNA damage promotes c-Abl translocation from nuclear to cytoplasm, where c-Abl promotes OTULIN phosphorylation at Tyr56. Then, OTULIN prompts the Wnt/β-catenin pathway by attenuating the linear ubiquitination of β-catenin, and facilitates breast cancer cells alteration to a chemoresistant state. Moreover, clinical data show that increased levels of OTULIN and β-catenin significantly correlate with poor prognosis and chemoresistance in TNBC patients [105].

According to the results of the viability of different breast cancer cell lines after cisplatin treatment, ER^-^ breast cancer is more resistant to cisplatin [106]. The deubiquitinase USP9X stabilizes MCL1, whose overexpression contributes to chemoresistance and poor prognosis in breast cancer [107]. Downregulation of USP9X reinforces cisplatin sensitivity in ER^-^ breast cancer cells, which is speculated to be a result of the degradation of MCL1 [106].

C-Jun activation domain-binding protein-1 (Jab1), also known as CSN5, which is negatively regulated and directly targeted by miR-17, increases cisplatin resistance in TNBC [108]. Jab1 also contributes to cellular resistance to cisplatin by enhancing Rad51 activity in DNA damage repair with the assistance of p53 [109].

EMT transcription factors are significant for the acquisition of chemoresistance in cancer cells. For example, radiation or chemotherapy induces the expression of the Snail/Slug family in ovarian cancers. This, in turn, enhances cell survival by weakening the expression of the p53-mediated apoptotic gene and derepressing the expression of selfrenewal genes [110]. Similarly, Snail1 may contribute to chemoresistance in breast cancer patients following the above-mentioned regulation. It has been demonstrated that USP27X is a putative deubiquitinase for Snail1, which enhances breast cancer cells resistance to cisplatin via stabilizing Snail1 and at least reinforcing repression of apoptosis associated genes [75].

### 5.4. PARPi

BRCA1/2 are key components in the process of homologous recombination (HR) targeting the repair of DNA double-strand breaks (DSBs). Additionally, Poly–(ADP–ribose) polymerase (PARP) functions as a critical enzyme for DNA single-strand breaks repair, making PARP inhibitors (PARPi) an effective therapeutic strategy for cancer patients with BRCA mutations [111]. Therefore, it is necessary to find valid biomarkers identifying breast cancers which are sensitive to PARPi treatment.

A study found that USP15 affects breast cancer cell sensitivity to PARPi via regulation of HR. MDC1 recruits USP15 to DNA damage sites, where the BRCT domain of BARD1 is deubiquitinated by USP15, thereby enhancing BRCA1/BARD1 retention that facilitates DSB end resection. Investigators also speculated that breast cancer patients with USP15 M861V and D967H mutants are more sensitive to PARPi treatment, suggesting that these two sites contribute to the interaction with BARD1 [112].

Moreover, BRCA2 recruits Rad51 to DSBs in the HR repair pathway to catalyze homologous pairing [113]. In addition, the deubiquitinase activity of UCHL3 is essential in this process. Mechanistically, ATM activates UCHL3 after DNA damage, which in turn enhances Rad51 interaction with BRCA2 via deubiquitination. Thus, UCHL3 strengthens the HR signaling pathway in DNA repair, rendering breast cancer cells resistant to PARPi. Likewise, according to clinical cases, UCHL3 overexpression functions as a prognostic index for unfavorable outcome in breast cancer patients [114].

RNF169 is an atypical regulator in DSB repair that augments the accurate HR pathway instead of the nonhomologous end joining (NEHJ) pathway [115]. USP7 interacts with RNF169 by UBL domains, then deubiquitinates and stabilizes RNF169, which effectively accumulates at DSBs in promotion of HR. As a result, the USP7–RNF169 axis contributes to accurate DSB repair, and facilitates breast cancer cells resistance to PARPi [116].

## 6. DUBs in Radioresistance and Radiosensitivity of Breast Cancer

Radiation therapy has increasingly become critical and conventional in breast cancer management. However, the presence of radioresistant cancer cells makes patients suffer from local tumor recurrences. It is therefore important to observe factors involve in radioresistance and explore potent tumor radiosensitizers.

It is well known that cancer stem cells (CSCs) are able to prompt cell cycle checkpoints, thus leading to radioresistance in tumors [117]. Meanwhile, EMT enables cells to obtain stem-like properties, indicating that EMT engages in radioresistance. Researchers identified that ZEB1, a core factor of EMT, is amplified in radioresistant subtypes of breast cancer. ZEB1 is phosphorylated by ATR, a component of the DNA damage repair (DDR) pathway. Then, ZEB1 combines with USP7 to increase its deubiquitinase and stabilization ability towards checkpoint kinase 1 (CHK1), thus facilitating the HR pathway that contributes to radioresistance [118]. In addition to EMT transcription factor ZEB1, long noncoding RNA *LINC02582* also promotes radioresistance through interacting with USP7 and stabilizing CHK1. *LINC02582* functionally serves as a molecular target of miR-200c, which has been previously demonstrated as a radiosensitizer in breast cancer [119]. PHF8 is also identified as another substrate of USP7, which involves in DSB repair via recruitment of BLM and KU70 [31]. In conclusion, interfering USP7 deubiquitinase activity elevates breast cancer sensitivity to radiation therapy.

Rad51, a component in DNA repair pathway, is regarded as a selective target to sensitize tumors to cytotoxic treatments [120]. It is found that UCHL3 weakens radiosensitivity in breast cancer cells by deubiquitinating and activating Rad51. Interventions targeting UCHL3 may improve the curative effect in combination with radiation treatment [114].

USP52 stabilizes the histone chaperone ASF1A by removing K48-linked polyubiquitin chains, then ASF1A delivers classical S-phase histones H3.1-H4 dimer to a replication-coupled chromatin. Therefore, USP52-mediated ASF1A deubiquitination is essential in sustaining genome stability upon DNA damage. Analysis of breast cancer cell viability showed that the USP52/ASF1A signaling promotes tumor cells resistance to ionizing radiation [121].

Moreover, the UCHL1/HIF-1 axis plays an important role in promoting breast cancer resistance to radiotherapy. UCHL1 upregulates the activity of HIF-1 via deubiquitination of its subunit HIF-1α. Then, HIF-1 activates reprogramming of glucose metabolism and the subsequent pentose phosphate pathway (PPP), thus increasing the level of reduced glutathione (GSH). It is widely recognized that intracellular antioxidants represented by GSH protect cancer cells from radiation-induced DNA lesions through scavenging free radicals and other oxidative products [122].

In summary, the ATM/ZEB1/USP7/CHK1, miR-200c/*LINC02582*/USP7/CHK1, USP7/PHF8, UCHL3/RAD51, USP52/ASF1A, and UCHL1/HIF-1 signaling axis are potential targets to improve the radiosensitivity of breast cancer.

## 7. Brief Summary

To sum up the above discoveries, DUB families involved in each step of breast tumorigenesis are summarized and shown in Table 1.

## 8. DUB Inhibitors for Breast Cancer Therapy

As previously described, DUBs play important roles in each stage of breast cancer progression. Therefore, targeting relevant DUBs and exploring potent DUB inhibitors appear to be potential therapeutic strategies for breast cancer patients (Table 2).

Researchers screened a small molecule NSC112200 targeting the cancer-promoting deubiquitinase ZRANB1 for pharmacological intervention. However, NSC112200 kills TNBC cells at micromolar concentrations and exhibits some toxicity in mouse models. Further experiments to identify a safer ZRANB1 inhibitor are needed clinically [49].

Curcumin, a CSN5 inhibitor, sensitizes breast cancer cells to immunotherapy by downregulating their PD-L1 expression [89]. In addition, researchers discovered a novel inhibitor, CSN5i-3, which significantly constrains tumor growth by suppressing proliferation and inducing G_1_ phase cell cycle arrest in breast cancer cells [123].

As FFA enhances TGF-β-promoted tumorigenesis through activation of ERK and USP9X, USP9X catalytic inhibitors such as WP1130 are regarded as effective treatments for obesity-related breast cancers [71]. WP1130 also increases the cytotoxic effects of cisplatin partly via its inhibitory effect on USP9X in ER^-^ breast cancer cells, suggesting combination therapy with WP1130 may be an efficient method for patients with ER^-^ breast cancer [106].

Perifosine is described as a novel UCHL3 inhibitor which is able to decrease the HR pathway in DSB repair by inducing ubiquitination of Rad51 and blocking Rad51 interaction with BRCA2. In breast cancer, perifosine considerably intensifies DNA lesions caused by PARPi such as Olaparib. Therefore, combination therapy of perifosine and PARPi is a promising strategy for Olaparib-resistant TNBC patients [124].

USP14, a key component that promotes breast tumorigenesis through its deubiquitination and stabilization of AR, is selectively inhibited by a small molecule, IU1 [99,125]. Cotreatment of the AR antagonist enzalutamide and IU1 downregulates AR and inhibits AR-related signaling pathways in breast cancer. Thus, IU1 in combination with enzalutamide offers an efficient strategy to suppress AR^+^ breast cancer growth [100].

Since USP1-mediated deubiquitination of KPNA2 contributes to tumorigenesis, researchers have found that inhibitors of USP1 such as pimozide and ML323 decrease breast cancer metastasis without showing obvious cytotoxicity in mice, offering a novel therapeutic target to be explored in the future [84].

Twist, a direct substrate of USP2, promotes tumor progression and drug resistance in TNBC. Consistently, it is documented that treatment with ML364, a USP2 inhibitor, attenuates migration and reinforces sensitivity to doxorubicin or paclitaxel in TNBC cells [79].

Since the two mechanisms for protein degradation in eukaryotic cells, the ubiquitin–proteasome pathway and the lysosomal pathway, are functionally coupled, combination therapy with deubiquitinase inhibitors and lysosomal inhibitors becomes an innovative approach for TNBC treatment. A report has confirmed that b-AP15 and RA-9, two novel inhibitors of the proteasomal 19S regulatory particle (RP) associated DUBs, including USP14 and UCHL5 [126,127], prompt autophagy in TNBC cells in response to UPS stress, and present synergistic inhibitory effects on tumor growth in conjunction with chloroquine, an FDA-approved drug inhibiting lysosomal degradation [128].

## Figures and Tables

**Table 1:** Roles of DUBs in breast cancer progression.

Family	DUB	Substrates	Effects	References
USP	USP7	ERα, PHF8, ECT2	promote tumor growth	[28,31,32]
		RNF169	promote chemoresistance	[116]
		PHF8, CHK1	promote radioresistance	[31,118]
	USP14	CyclinB1	promote tumor growth	[24,25]
		AR	promote chemoresistance	[99,100]
	USP22	H2B, c-Myc, CCND1, ERα	promote tumor growth	[8,18,21,22]
		c-Myc	promote tumor metastasis	[8,80]
		ERα	promote chemoresistance	[22]
	USP36	c-Myc	promote tumor growth	[9]
	USP15	BMI1	promote tumor growth	[34]
		BMI1, TβRI	promote tumor metastasis	[34,66]
		BARD1	promote chemoresistance	[112]
	USP44	H2B	promote or suppress tumor growth	[19]
	USP3	KLF5	promote tumor growth	[13]
	USP4	PDCD4, HDAC2	promote or suppress tumor growth	[39,40]
		TβRI	promote tumor metastasis	[65]
	USP9X	RNF115, BCL9, CEP131, YAP1, LATS	promote or suppress tumor growth	[41,42,45,46,47,48]
		RNF115, BCL9, SMAD4	promote tumor metastasis	[42,45,71]
		ERα cofactors, MCL1	promote chemoresistance	[94,107]
	USP21	FOXM1	promote tumor growth	[59]
	USP28	LSD1	promote tumor growth	[61]
	CYLD	NEMO and upstream regulatory factors of IKK	suppress tumor growth	[51,52]
	USP27X	Snail1	promote tumor metastasis	[75]
		Snail1	promote chemoresistance	[75]
	USP20	SNAI2	promote tumor metastasis	[76]
	USP51	ZEB1	promote tumor metastasis	[77]
	USP2	BMI1	promote tumor metastasis	[79]
	USP1	KPNA2	promote tumor metastasis	[84]
		ERα	promote chemoresistance	[93]
	USP52	ASF1A	promote radioresistance	[121]
OTU	OTUD3	p53, PTEN	suppress tumor growth	[37,38]
	ZRANB1	EZH2	promote tumor growth	[49]
	OTUD1	SMAD7	suppress tumor metastasis	[69]
	OTUB1	PD-L1	promote immune escape	[91]
	OTULIN	β-catenin	promote chemoresistance	[105]
JAMM	PSMD2	p21, p27	promote tumor growth	[57]
	EIF3H	YAP	promote tumor metastasis	[83]
	Jab1/CSN5	PD-L1	promote immune escape	[89]
		Rad51	promote chemoresistance	[108,109]
MJD	ATXN3L	KLF5	promote tumor growth	[14]
	ATXN3	KLF4	promote tumor metastasis	[82]
UCH	BAP1	KLF5	promote tumor growth	[15]
		KLF5	promote tumor metastasis	[15]
	UCHL1	TβRI, HIF-1	promote tumor metastasis	[67,85]
	UCHL3	Rad51	promote chemoresistance	[114]
		Rad51	promote radioresistance	[114]

**Table 2:** DUB inhibitors as potential agents for breast cancer progression.

Family	DUB	Inhibitor	Structure	References
USP	USP14	IU1	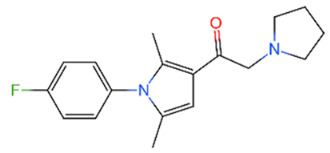	[99,100,125,129]
	USP9X	WP1130	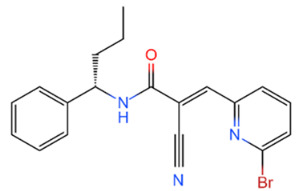	[71,106,130]
	USP2	ML364	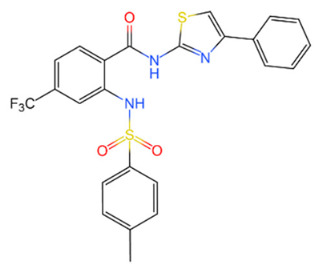	[79,131]
	USP1	Pimozide	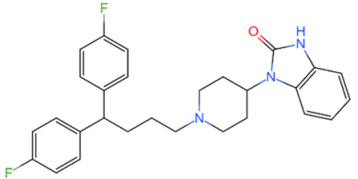	[84,132]
		ML323	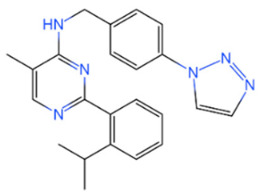	[84,133]
	ZRANB1	NSC112200	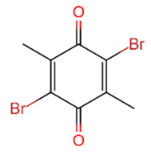	[49,134]
JAMM	Jab1/CSN5	Curcumin	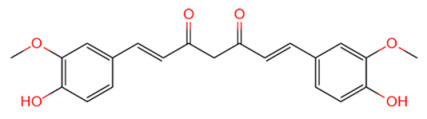	[89,135]
		CSN5i-3	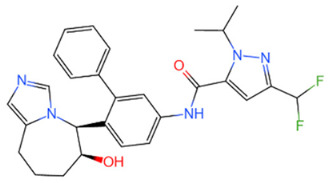	[123,136]
MJD	UCHL3	Perifosine	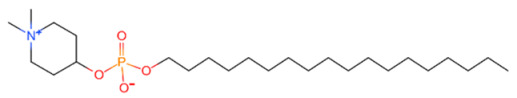	[124,137]
USP, UCH, etc.	19S RP DUBs	RA-9	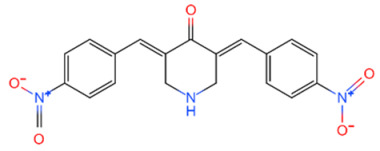	[128,138]
		b-AP15	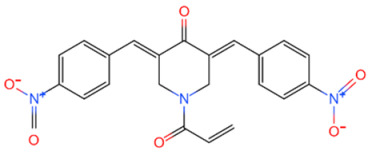	[128,138]

## Data Availability

Not applicable.
